# Supplementary material for: Deep mutational scanning of H5 hemagglutinin to inform influenza virus surveillance
Source: PLoS Biol. 2024 Nov 12;22(11):e3002916. doi: 10.1371/journal.pbio.3002916 (PMC11584116; doi:10.1371/journal.pbio.3002916)
Supplement: Peer review history

# Editor report: Decision Letter 0

**DOI:** 10.1371/journal.pbio.3002916.r001

**Authors:** Nonia Pariente, PhD

24 Oct 2024

Dear Jesse, 

Thank you for submitting your manuscript entitled "Deep mutational scanning of H5 hemagglutinin to inform influenza virus surveillance" for consideration in PLOS Biology.

We have now had the time to assess your manuscript, your responses to the prior peer-review reports and the revisions made, as well as to discuss the file with an academic editor with relevant expertise. We consider that the revision has addressed the reviewer concerns well and we would like to publish your work. 

However, before we can proceed, we need you to complete your submission in Editorial Manager by providing some metadata, and edit your manuscript so that it comply with our editorial policies (detailed below). To this end, please login to Editorial Manager where you will find the paper in the 'Submissions Needing Revisions' folder on your homepage. Please click 'Revise Submission' from the Action Links, complete (or edit) all additional questions in the submission questionnaire, and replace submission files, as needed.

a) Please confirm that the following financial statements are not missing the grant number: Gift from Open Philanthropy (to N.P.K.), UK Medical Research Council/Department for Environment (J.Y., Y.L., T.P.P), Food and Rural Affairs (Defra, UK) (J.Y., Y.L., T.P.P), and Howard Hughes Medical Institute (J.D.B.).

b) Thank you for providing the protocol number for the ferret experiments, please also include the protocol number for the murine experiments. Additionally, the Ethics statement needs to be a separate, independent (and the first) subheading in the Material & Methods section. You currently have it after “Serum generation”; please provide it separately and place it at the beginning of the Material & Methods section.

c) You may be aware of the PLOS Data Policy, which requires that all data be made available without restriction: http://journals.plos.org/plosbiology/s/data-availability. For more information, please also see this editorial: http://dx.doi.org/10.1371/journal.pbio.1001797

Please supply the numerical values either in the a supplementary file or as a permanent DOI’d deposition for the following figures:

Figure 2AC, 3A, 4CD, 5AD, 6CD, S1D, S2BD, S3AB, S4, S5ABC, S7, S8, S9C, S10ABC

NOTE: the numerical data provided should include all replicates AND the way in which the plotted mean and errors were derived (it should not present only the mean/average values).

e) Please provide the tree files for Fig. 1B, 6AB, S9AB

f) Please indicate in all relevant main and supplementary Figure legends where the data can be found, e.g. “The data underlying this Figure can be found in S1 Data” or “The data underlying this Figure can be found in https://doi.org/10.5281/zenodo.XXXXX”

g) Please ensure that your Data Statement in the Editorial Manager submission system accurately describes where your data can be found and is in final format, as it will be published as written there.

h) Thank you for providing the code and additional information like plasmid maps on Github. However, per journal policy, we cannot accept sole deposition of code in GitHub, as this does not provide a DOI and can be silently changed after after publication. You can archive this version of your publicly available GitHub code and additional data in Zenodo. Once you do this, it will generate a DOI number, which you will need to provide in the Data Accessibility Statement (you are welcome to also provide the GitHub access information, as this may be useful for readers as well). See the process for doing this here: https://docs.github.com/en/repositories/archiving-a-github-repository/referencing-and-citing-content

Once your full submission is complete, your paper will undergo a series of checks by our journal Operations team and it will be sent to me. If the above editorial requests have been addressed, I will be able to issue an "accept in principle" decision and your manuscript will enter our production process. 

To provide the metadata for your submission and address the request above, please Login to Editorial Manager (https://www.editorialmanager.com/pbiology) by next Monday 28 October. Please let me know if this process is likely to take longer.

With best wishes,

Nonia

Nonia Pariente, PhD, 

Editor-in-Chief

PLOS Biology

npariente@plos.org

# Editor report: Decision Letter 1

**DOI:** 10.1371/journal.pbio.3002916.r002

**Authors:** Nonia Pariente, PhD

29 Oct 2024

Dear Jesse,

Thank you for the submission of your revised Research Article "Deep mutational scanning of H5 hemagglutinin to inform influenza virus surveillance" for publication in PLOS Biology. On behalf of my colleagues and the Academic Editor, Katherine Kedzierska, I am pleased to say that we can in principle accept your manuscript for publication, provided you address any remaining formatting and reporting issues. These will be detailed in an email you should receive within 2-3 business days from our colleagues in the journal operations team; no action is required from you until then. Please note that we will not be able to formally accept your manuscript and schedule it for publication until you have completed any requested changes.

Please take a minute to log into Editorial Manager at http://www.editorialmanager.com/pbiology/, click the "Update My Information" link at the top of the page, and update your user information to ensure an efficient production process.

PRESS

We frequently collaborate with press offices. If your institution or institutions have a press office, please notify them about your upcoming paper at this point, to enable them to help maximise its impact. If the press office is planning to promote your findings, we would be grateful if they could coordinate with biologypress@plos.org. If you have previously opted in to the early version process, we ask that you notify us immediately of any press plans so that we may opt out on your behalf.

We also ask that you take this opportunity to read our Embargo Policy regarding the discussion, promotion and media coverage of work that is yet to be published by PLOS. As your manuscript is not yet published, it is bound by the conditions of our Embargo Policy. Please be aware that this policy is in place both to ensure that any press coverage of your article is fully substantiated and to provide a direct link between such coverage and the published work. For full details of our Embargo Policy, please visit http://www.plos.org/about/media-inquiries/embargo-policy/.

Thank you again for choosing PLOS Biology for publication and supporting Open Access publishing. We look forward to publishing your study. 

Best,

Nonia

Nonia Pariente, PhD, 

Editor-in-Chief

PLOS Biology

npariente@plos.org
